# Names Again

**Published:** 1869-08-15

**Authors:** 


***Names Again.***

There is no more annoying circumstance than similarity of names in a large city like Chicago and San Francisco. Our contemporary of the *Pacific Medical Journal* is under constant tribulation from an assumed namesake, who sells *Patent French Safes!* and runs a quack infirmary especially for the convenience of veterans wounded in venereal wars. In this city our colleague, Prof. Moses Gunn, whose reputation both as a teacher and surgeon has been won by nearly a quarter of a century of toil and vast experience, is every day confounded with a new comer, perhaps of the same name, who has gained a handle to his cognomen by adroit connection with a so-called *Eclectic* concern recently hatched by the sapient legislators of Illinois, in anticipation probably, of the good time coming when none but M.Ds. can practice in this commonwealth. A rose by any other name will smell as sweet—why not a quack doctor?

Ludicrously enough, in one sense, if it were not for the trouble to which it subjects patients, the new city directory, *probably* again under the influence of some adroit manipulation, puts down the residence only, of Prof. Gunn, several miles distant from his office, which is at Rush Medical College, and labels him "physician" only. The namsake, *perhaps*, is given office and residence both, and duly labelled "physician and surgeon."

We deem it a duty we owe the profession of the north-west, that they should be put on their guard against this ingenious swindle. It is due to Prof. Gunn to state that he has not instigated the present notice. His office is at Rush Medical College, and physicians and patients should "govern themselves accordingly."

What the present editorial writer has suffered from a somewhat similar confusion of names need not here be put down. He has at least the consolation that some of his "short comings" have been set down, both by the public and the Recording Angel, to the account of "*the other man.*"

